# Prenatal diagnosis of a “living” oropharyngeal fetus in fetu: a case report

**DOI:** 10.1186/s12884-019-2612-0

**Published:** 2019-11-29

**Authors:** Ling Wang, Baiguo Long, Qichang Zhou, Shi Zeng

**Affiliations:** 1 Department of Ultrasound Diagnosis, Maternal and Child Health Center of Zhuzhou, 128 Station Road, Zhuzhou, Hunan China; 2 Department of Ultrasound Diagnosis, The Second Xiangya Hospital of Central South University, 139 Renmin Road (M), Changsha, 410011 Hunan China

**Keywords:** Fetus in fetu, Prenatal, Oropharyngeal

## Abstract

**Backgroud:**

Fetus in fetu (FIF) is a rare malformation in which a parasitic twin within its more mature twin. Most of the FIF locate in the retroperitoneum and are acardiac and anencephalic.

**Case presentation:**

Here, we describe a unique case of oropharyngeal fetus in fetu with a rudimentary two-chambered heart detected by prenatal ultrasonography. The parents terminated this pregnancy after counseling. Macroscopic examination found a solid mass between the oral and fetal chest, with a rudimentary two-chambered heart at the lowest part of the mass. Microscopic findings showed amniotic membrane, skin, cartilage, gastrointestinal and neural tissue.

**Conclusions:**

Prenatal ultrasound can identify rudimentary organs suspecting FIF from early pregnancy. Detection of fetal heart beat facilitates differential diagnosis with teratomas, providing essential information for parental consulting and management.

## Introduction

Fetus in fetu (FIF), fetiform masses enclosed within the body of the relatively normal fetus, is the one of internal forms of parasitic twin [1]. As reported initially in 1935 [2], FIF was described as “as a mass containing a vertebral axis often associated with other organs around this axis”. With the increased reports and advanced knowledge, the presence of spinal column wasn’t the necessity for diagnosis any more. The characteristics of FIF include a mass enclosed within a distinct fibrous membrane, partially or completely covered by normal skin, grossly recognizable anatomic structures and supplied by a relatively large blood vessel from the host fetus [1, 3, 4]. Moreover, the presence of grossly recognizable anatomic structures is the key point for differentiating FIF from another internal form of parasitic twin- teratoma. A teratoma, on the other hand, is germ cell tumor without high level of organization and unable to develop mature tissue [1]. Complete surgical resection of the mass is the main management for FIF. The prognosis of FIF is generally favorable based on its typical benign feature although there was an atypical potentially malignant case reported [5].

FIF is a rare malformation, generally acardiac and anencephalic, with frequent localization in the retroperitoneum. Here, we describe a unique case of oropharyngeal fetus in fetu with a rudimentary two-chambered heart detected. Prenatal ultrasound is a useful tool to identify well developed organs in the FIF, evaluate the size and spatial relationship of the mass, and help to guide the ex utero intrapartum treatment procedure for newborn FIF [6, 7], or offer the reasonable option of early termination for severe cases.

## Case presentation

**Fig. 1:**
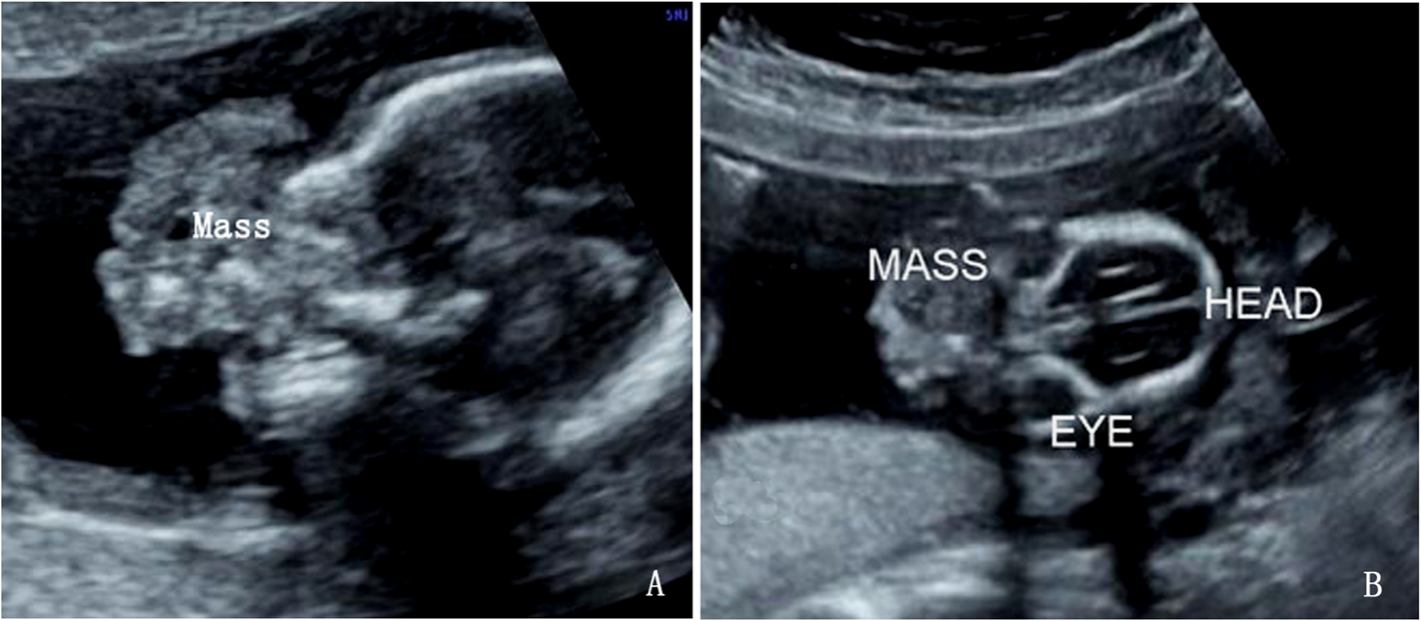
A nonhomogeneous mass, protruding out of the mouth, was identified in the fetus oropharyngeal section. **a** sagittal view; **b** coronal view

**Fig. 2:**
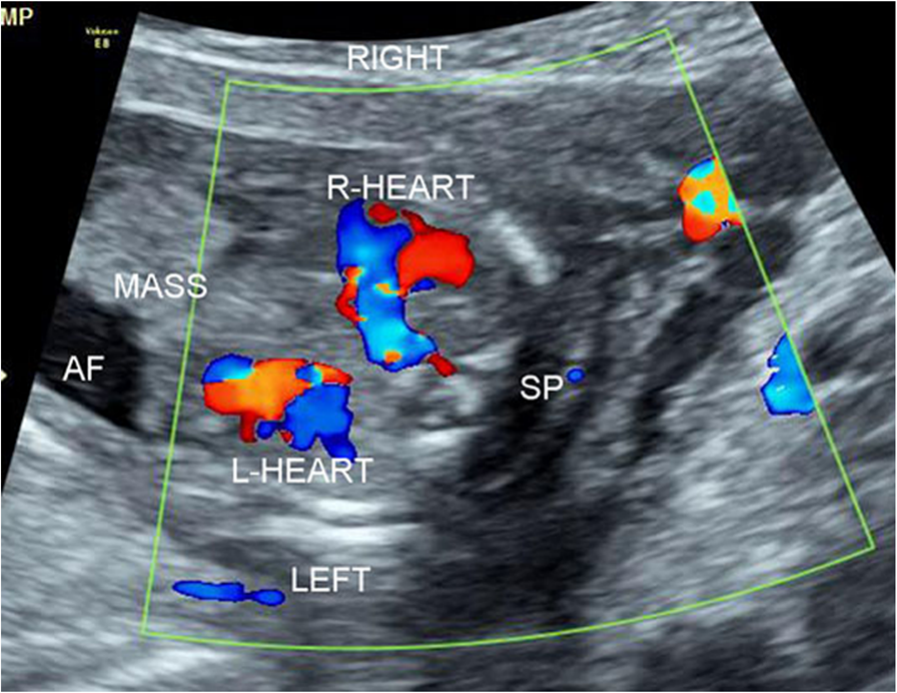
Color Doppler demonstrated pulsive color signals in the heart-like structure at the lowest end of the mass within the fetal left chest. The heart of the fetus was demonstrated on the right chest. AF, amniotic fluid; Sp, spine

**Fig. 3:**
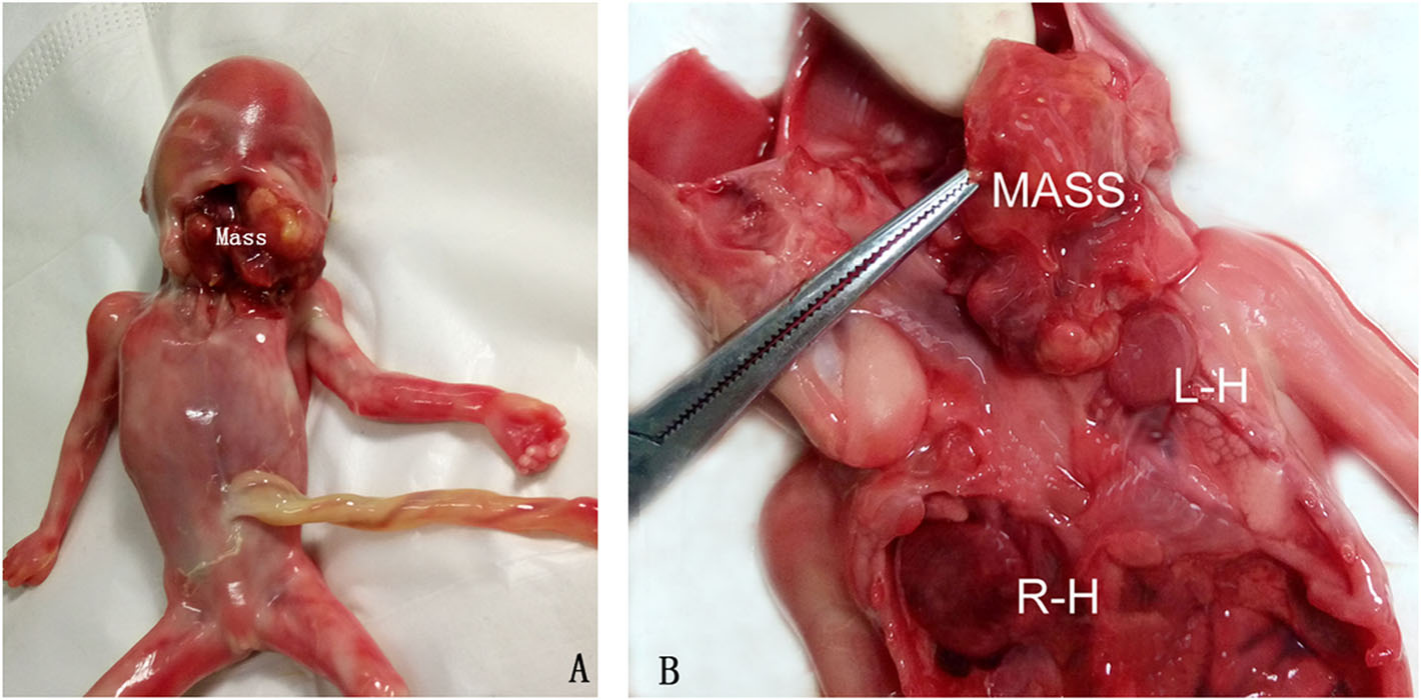
Macroscopic examination found a solid mass between the oral and fetal chest (3A), with a rudimentary two-chambered heart at the lowest part of the mass (3B).L-H, left heart; R-H, right heart

A 28-year-old, gravida 1 para 0 woman was referred for routine obstetric ultrasound scan at 16 weeks’ gestation. The family history was negative for congenital malformations, and there was no history of medication and drug use during pregnancy. The biometry measurements were appropriate for gestation age. A nonhomogeneous mass, protruding out of the mouth, was identified in the fetus oropharyngeal section (Fig. 1). The mass was about 32 mm × 27 mm × 28 mm, with the lower part locating in the fetal chest and upper part locating in fetal oral cavity. A two-chamber beating heart-like structure was demonstrated at the lowest end of the mass within the fetal left chest. Doppler demonstrated pulsatile color signals in the heart-like structure (Fig. 2). The heart of the fetus was demonstrated on the right chest. Multiple calcifications were also noted in the mass. Besides, the hard palate and superior alveolar bone were absent. The amniotic fluid index and umbilical artery pulsatility index were normal. Fetus in fetu was suspected considering the heart-like structure in the mass. The parents terminated this pregnancy after counseling. The fetal karyotype was 46,XX. Macroscopic examination found a solid mass between the oral and fetal chest, with a rudimentary two-chambered heart at the lowest part of the mass (Fig. 3). Microscopic findings showed amniotic membrane, skin, cartilage, gastrointestinal and neural tissue.

## Discussion and conclusions

The presence of well differentiated organogenesis is the key feature in the FIF. Vertebral column and limbs were the most recognizable structures in FIF. Skin, intestinal, bones, cartilage, lung, and adrenal tissue were also often present. A review [4] of reported 32 prenatal cases of FIF concluded that 90% of cases had a vertebral column, 90% had recognize bony extremity or rudimentary limb, 60% had central nervous tissue, 26% had gastrointestinal tissue, and 20% had respiratory tissue. All of reported FIF were acardiac except one case which found rudimentary heart structure by postnatal ultrasound [6]. To date, the present case is the first FIF with cardiac structure detected prenatally. Actually, except for the heart structure, there was no obvious vertebral column, extremity limb and bone or other recognizable organs in our case. The presence of two chamber heart in the mass was the important clue for the diagnosis of FIF in this case and was confirmed by autopsy.

FIF mostly located within the abdomen (80%), skull (8%) and sacral region (8%) [4]. The location of oral/ oropharyngeal was only reported in two prenatal cases [7, 8]. Although the mass in FIF was benign, it may compress surrounding tissues and cause destructive lesions and maldevelopment of surrounding organs. In this case, the hard palate and superior alveolar bone were absent which may related to the existence of FIF. Lindsey M’s review reported 97% of FIF had a good prognosis after complete surgical resection of the parasitic twin [9]. Few adverse outcomes were reported. One patient with intracranial FIF was alive during follow-up but suffered poor muscle tone and underwent ventriculoperitoneal shunt [10]. One cases with multiple abdominal FIF died on postoperative day 32 due to hydrops [11]. Malignant recur as a yolk sac tumor was reported after retroperitoneal FIF resection [5]. We couldn’t conclude that there was relation between outcome and the site of implant based on the limited cases. However, it is certain that the complication varied among the different locations. For abdominal FIF, abdomen distention, emesis and peritoneal inflammation may be associated. For cranial FIF, obstructive hydrocephalus and mental retardation may be associated. For thoracic FIF, dysphagia and even airway obstruction may occur and therefore emergency surgery is needed [7, 8]. Also, for FIF with multiple or large mass, or with abundant blood supply, heart failure and hydrops is the associated risk.

With the advance of acoustics technology, ultrasound could be used to detect such malformation in utero. Most of cases with FIF were detected in 2nd and 3rd trimester [4]. Our case was demonstrated at 16 weeks’ gestation, the most early gestation age reported so far. We may hypothesize that in severe cases like the one presented here, the condition may be suspected in the first trimester at the time of first trimester screening, if such essential examination is performed and if trained operators are involved. Prenatal ultrasound can identify rudimentary organs suspecting FIF from early pregnancy. Detection of fetal heart beat facilitates differential diagnosis with teratomas, providing essential information for parental consulting and management..

## Data Availability

The datasets used and/or analysed during the current study are available from the corresponding author on reasonable request.

## Abbreviation

FIF: Fetus in fetu
